# Environmental Performance of a Waste-Derived Phenol–Formaldehyde/Glass-Fibre Composite: Leaching and Multispecies Evidence for Safe Circular Design

**DOI:** 10.3390/polym18212744

**Published:** 2026-11-04

**Authors:** Agnieszka Podolak, Justyna Koc-Jurczyk, Zuzanna Sylwestrzak, Łukasz Jurczyk, Waldemar Prokop

**Affiliations:** 1 Institute of Agricultural Sciences, Environment Protection and Management, Faculty of Technology and Natural Sciences, University of Rzeszów, Ćwiklińskiej 1a, 35-601 Rzeszów, Poland; jkoc@ur.edu.pl (J.K.-J.); ljurczyk@ur.edu.pl (Ł.J.); 2 Faculty of Oceanography and Geography, University of Gdańsk, Piłsudskiego 46, 81-378 Gdynia, Poland; zuzanna.sylwestrzak@ug.edu.pl; 3 C4IN, Rudna Wielka 27d, 36-054 Rudna Wielka, Poland; wprokop@interia.pl; 4 PureEkoTech Sp. z o.o., Metalowcowa 26, 39-460 Nowa Deba, Poland

**Keywords:** sustainable polymer composites, industrial waste valorisation, leaching behaviour, ecotoxicity, circular design

## Abstract

Industrial-residue valorisation through polymer-composite manufacturing can reduce reliance on virgin resources, yet the environmental safety of such materials is rarely evaluated beyond conventional physicochemical characterisation. This study provides a novel, integrated assessment of a waste-derived phenol–formaldehyde/glass-fibre composite by combining pH-dependent leaching analysis with a multispecies ecotoxicological test battery. The composite contained post-production glass fibres, paper flakes, and viscose fibres bound with phenol–formaldehyde resin. Under near-neutral extraction conditions, most analysed metals and metalloids showed limited release. In contrast, acidic conditions substantially increased the mobilisation of Ba, Cr, Zn, Cu, and Pb, while the phenol index increased approximately 28-fold, from 0.25 ± 0.06 to 6.9 ± 1.7 mg L^−1^. Biological responses differed markedly among taxa. *Hordeum vulgare* and *Eisenia andrei* showed limited short-term effects, whereas the marine diatom *Skeletonema marinoi* exhibited pronounced concentration- and time-related inhibition, with no cells detected after three days of exposure to the highest extract loading. The *Aliivibrio fischeri* assay also revealed concentration-dependent acute toxicity, with the strongest effects observed during the initial exposure period. The pronounced algal response could not be fully explained by the targeted chemical analyses, indicating a possible contribution of unidentified leachable constituents or mixture effects. The novelty of this study lies in demonstrating that technical functionality, waste diversion, and low concentrations of selected regulated contaminants do not alone ensure the environmental safety of waste-derived polymer composites. Integrating stress-condition leaching with sensitive biological endpoints offers a practical framework for safe-by-design formulation, application selection, and end-of-life management. Further studies should address material ageing, repeated leaching, particle release, and non-target identification of bioactive constituents.

## 1. Introduction

Increasing anthropogenic pressure accelerates environmental degradation through the consumption of natural resources, the generation of industrial residues, and the accumulation of pollutants in environmental compartments [1]. These challenges have intensified the search for material-development strategies that reduce dependence on virgin resources while promoting the recovery and reuse of by-products generated by industrial processes [2]. At the same time, modern applications increasingly require materials that combine high mechanical performance, durability, and functional versatility with a reduced environmental burden [3]. Meeting these expectations requires not only the substitution of primary raw materials with secondary resources, but also evidence that newly developed materials remain environmentally acceptable throughout their intended use and at end-of-life.

Polymer composites reinforced with glass, carbon, or other fibres are widely used in construction, transport, infrastructure, and consumer products because of their favourable strength-to-weight ratio, durability, chemical resistance, and design flexibility. Appropriate selection of the matrix and reinforcement can produce materials that are lighter, mechanically robust, cost-effective, and, in some applications, associated with a lower environmental footprint than conventional alternatives [4,5]. The incorporation of industrial residues into hybrid composites offers an additional opportunity to reduce the consumption of virgin resources and recover materials that might otherwise be landfilled or incinerated. Waste-derived composites can therefore provide alternatives to conventional plastics, metals, ceramics, and polymer composites based solely on primary constituents [6,7].

The use of glass fibres, polymer residues, paper, sawdust, ash, and other secondary materials in composite manufacturing is consistent with circular-economy principles, particularly the retention of material value and the extension of resource circulation [8]. However, the diversion of waste from disposal does not, by itself, demonstrate that the resulting composite is environmentally sustainable. Circularity claims should also consider the chemical stability of the material, the potential release of contaminants during contact with water, and the biological effects of leachable constituents under realistic use, ageing, and end-of-life conditions.

Most studies on waste-derived polymer composites focus primarily on manufacturing optimisation, mechanical performance, structural integrity, thermal behaviour, and functional applicability. In contrast, their environmental interactions and ecotoxicological profiles are considerably less well characterised. Physicochemical analyses can identify selected contaminants and quantify their release under defined conditions, but they may not capture the combined biological activity of complex mixtures, unidentified compounds, degradation products, or low-molecular-weight fractions released from polymer matrices. Consequently, the evaluation of environmental safety should complement conventional material characterisation with biological testing capable of detecting effects that cannot be predicted from targeted chemical data alone [9].

Ecotoxicological bioassays provide an effect-based approach to environmental assessment and are particularly useful when the composition of a leachate is complex or only partially characterised. Standardised short-term tests can rapidly identify adverse responses across organisms representing different trophic levels and ecological compartments, thereby supporting preliminary hazard identification and early-stage material screening [10]. Although acute toxicity tests do not reproduce long-term environmental exposure, ageing, bioaccumulation, or chronic effects, they constitute an important first step in a broader environmental-risk assessment by identifying immediate and organism-specific responses [11].

The present study investigated the environmental safety of a waste-derived phenol–formaldehyde/glass-fibre composite manufactured from post-production glass fibres, paper flakes, and viscose fibres bound with phenol–formaldehyde resin. The main objective was to assess potential environmental hazards associated with constituents released from the composite by characterising its leachates and evaluating their ecotoxicological effects on aquatic and terrestrial organisms. Specifically, the study aimed to determine whether aqueous extracts of the composite induced concentration-dependent and taxon-specific biological responses, whether extraction under near- neutral conditions adequately reflected the potential mobility of chemical constituents, and whether acidic conditions enhanced the mobilisation of metals and phenolic compounds. To achieve these objectives, targeted chemical characterisation and pH-dependent leaching analysis were combined with a multispecies ecotoxicological test battery involving organisms from aquatic and terrestrial environments. It was hypothesised that the composite would exhibit limited contaminant mobility and low acute toxicity under near-neutral screening conditions, whereas biological responses would differ among test organisms and increase with extract concentration. The novelty of this study lies in integrating pH-dependent chemical leaching with ecotoxicological responses of organisms representing different trophic levels and environmental compartments. By extending the assessment beyond waste utilisation and technical performance, the study addresses the potential release of biologically active constituents from a waste-derived polymer composite under different exposure conditions. This integrated approach provides evidence relevant to safe-by-design material development, the selection of appropriate application, and the environmental assessment of end-of-life management options for recycled polymer composites.

## 2. Materials and Methods

### 2.1. Composite Board Aqueous Extracts

**Figure 1:**
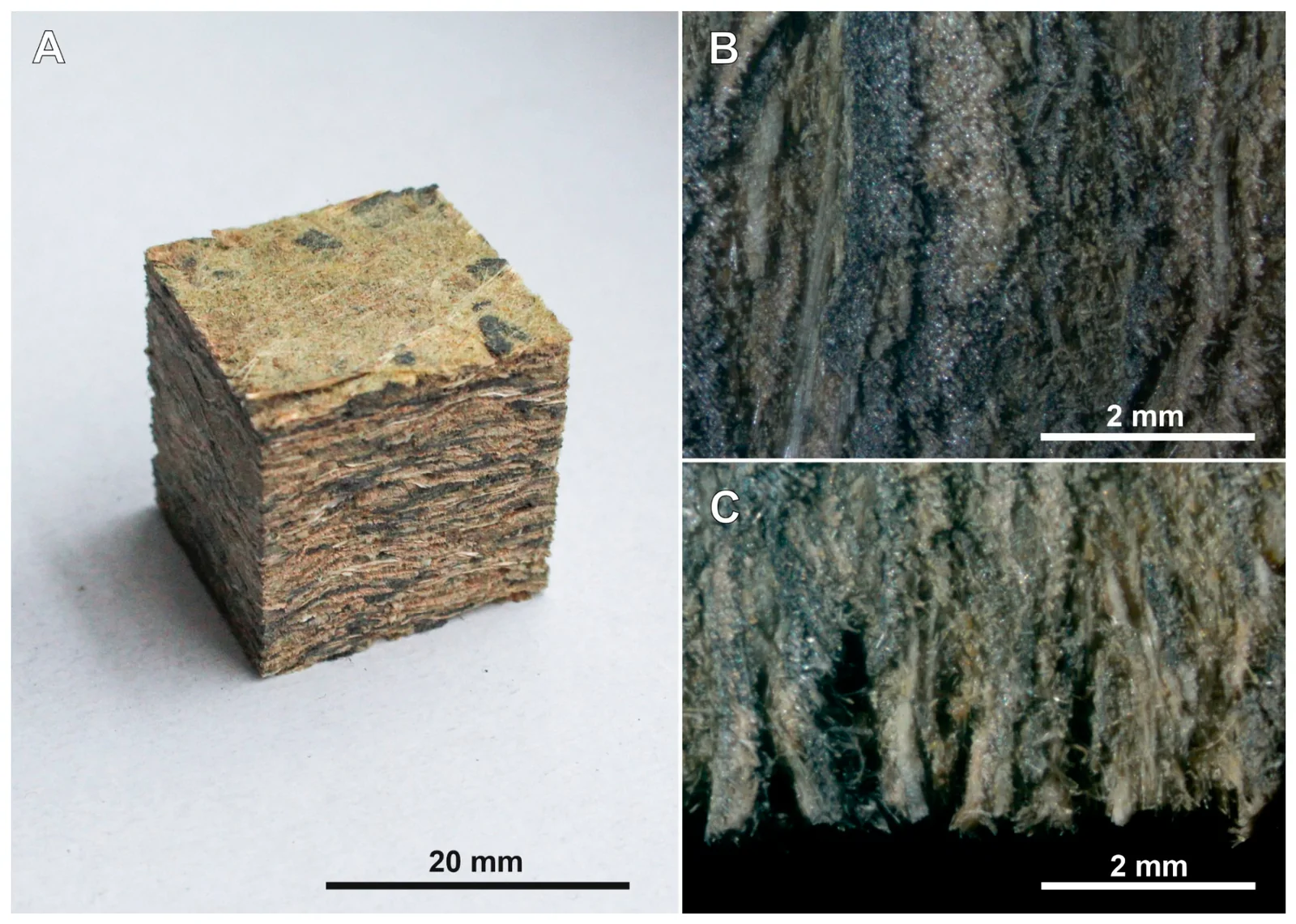
Waste-derived phenol–formaldehyde/glass-fibre composite board: (**A**) macroscopic specimen; (**B**) surface view and (**C**) exposed cross-section.

The investigated composite board was manufactured predominantly from post-production residues, with phenol–formaldehyde resin added as a virgin binding component. According to the production formulation provided by the manufacturer, the final composite consisted of 70 wt.% glass fibres, 20 wt.% phenol–formaldehyde resin, 7 wt.% paper flakes, and 3 wt.% viscose fibres. The glass fibres and phenol–formaldehyde resin were introduced as separate components during manufacturing. Prior to board formation, the solid waste-derived components were mechanically fragmented and dry-mixed, after which the phenol–formaldehyde resin was added as the binding phase. The resulting mixture was formed into boards by hot pressing. The panels were approximately 20 mm thick and had a nominal density of 1100 kg m^−3^, with a manufacturer-specified production tolerance of ±10%. According to the manufacturer’s technical datasheet for this product type, the minimum bending strength was 25 MPa, and the elastic modulus was at least 3000 MPa. After 24 h of water exposure, thickness swelling did not exceed 4%, while water absorption remained below 5%. The thermal conductivity coefficient was 0.3 W m^−1^ K^−1^. The material was classified as B-s2,d0 in accordance with EN 13501-1:2018 [12]. Detailed processing parameters, including fibre dimensions, pressing temperature, pressure, and curing time, were not disclosed by the manufacturer because preparation of a patent application for the manufacturing process is currently in progress. The reported composition, general manufacturing sequence, and technical properties were supplied by the manufacturer and were not independently verified in the present study. The composite boards used in the experiments were obtained from several production batches supplied by an anonymous industrial manufacturer (Figure 1).

The composite boards were cut into uniform cubes measuring approximately 2 × 2 × 2 cm (approximately 8 cm^3^). To remove loose particles generated during cutting, the specimens were rinsed once with 1 L of distilled water and then air-dried at room temperature for seven days to minimise the influence of residual moisture. The aqueous extracts used for physicochemical analyses were prepared by the laboratory in glass vessels using deionised water at a liquid-to-solid ratio of 10 L kg^−1^, corresponding to a nominal material concentration of 100 g L^−1^. The extraction procedure was adapted from PN-EN 12457-4:2006 [13] because the dimensions of the composite cubes exceeded the particle-size range specified in the standard. The vessels were closed and agitated at room temperature for 24 h at 150 rpm using a laboratory shaker. Following extraction, the eluates were filtered by gravity, without vacuum, through Whatman No. 1 filter paper. Composite specimens originating from several production batches were used.

Physicochemical analyses were performed by external laboratories using validated analytical procedures. Total organic carbon (TOC) in the solid composite was determined according to PN-EN 15936:2022-07 [14]. Polycyclic aromatic hydrocarbons (PAHs) were analysed using the laboratory procedure PB/I/6/F:10.04.2017 [15], volatile aromatic hydrocarbons (BTEX) according to PN-ISO 14507:2007 [16] and laboratory procedure PB/I/3/G:10.04.2017 [17], and polychlorinated biphenyls (PCBs) according to laboratory procedure PB/I/39/B:10.04.2017 [18].

In the aqueous extracts, chloride, sulfate, and fluoride contents were determined according to PN-EN ISO 10304-1:2009/AC:2012 [19], dissolved organic carbon (DOC) according to PN-EN 1484:1999 [20], and metals and metalloids according to PN-EN ISO 17294-2:2016-11 [21]. Mercury was determined using laboratory procedure PB-LL-20.2016 [22], whereas total dissolved solids (TDSs) were determined according to PN-EN 15216:2022-03 [23]. The phenol index/volatile phenols were determined according to PN-EN ISO 14402:2004 [24]. Separate aqueous extracts were prepared using deionised water with an initial pH of approximately 6.0 and 3.5.

### 2.2. Algal Growth Inhibition Test

The algal growth inhibition test was performed using the marine diatom *Skeletonema marinoi* [25], strain BA98, isolated from coastal waters of the southern Baltic Sea and maintained as a unialgal culture in the Culture Collection of Baltic Algae (CCBA), University of Gdańsk, Poland.

Three aqueous extracts of the composite material were tested. The extracts were prepared independently at nominal material concentrations of 50, 100, and 150 g L^−1^, corresponding to different liquid-to-solid ratios. Each treatment was tested in three independent culture flasks. The control consisted of F/2 culture medium prepared according to Guillard [26]. Nutrient components required for algal growth were present in the exposure media; however, their exact proportions in the individual extract treatments were not recorded.

The cultures were prepared in F/2 medium adjusted to a salinity of 8 PSU and inoculated with *S. marinoi* in the logarithmic growth phase at an initial density of 100,000 ± 500 cells mL^−1^. The exposure period lasted 3 days. Cultures were maintained at 18 °C under a 16:8 h light–dark photoperiod and a photosynthetically active radiation intensity of 60 µmol photons m^−2^ s^−1^, provided by OSRAM L 36W/640-1 lamps.

Cell abundance was determined after 1, 2, and 3 days of exposure using a Nikon Eclipse 80i light microscope equipped with a Nikon DS-U2 digital camera (Nikon Corporation, Tokyo, Japan) at 400× and 600× magnification. The pH and salinity of the control and exposure media were measured before and after the exposure period (ELMETRON, Zabrze, Poland). Cell abundance was expressed relative to the initial abundance recorded at the start of the experiment. Results are presented as mean values with variability calculated from three independent culture flasks per treatment.

### 2.3. Seed Germination and Early Seedling Growth Test

Certified, untreated seeds of *Hordeum vulgare* L. (Poaceae) were used to assess germination and early seedling growth. Before the experiment, seeds were visually selected to ensure a similar size and the absence of visible damage. Initial seed viability was at least 95%, as confirmed in preliminary control tests.

Three independently prepared aqueous extracts of the composite material were tested at nominal concentrations of 50, 100, and 150 g L^−1^. For each treatment, a total of 100 seeds were distributed among three independent 10 cm glass Petri dishes, with 33, 33, and 34 seeds per dish, respectively. Each dish was lined with Whatman No. 1 filter paper and moistened with 15 mL of the respective undiluted extract. The negative control was prepared in the same manner using distilled water, with three independent Petri dishes and the same number of seeds and liquid volume.

The Petri dishes were covered with lids and secured with elastic bands to prevent the developing seedlings from displacing the lids. The test was conducted for 72 h at 25 °C in continuous darkness using a procedure adapted from ISO 18763:2016 [27]. After exposure, seedling growth was stopped by freezing the samples at −18 °C for 24 h. Following thawing, the number of germinated seeds was recorded, and root and shoot lengths were measured manually with a ruler to the nearest 1 mm.

A seed was considered germinated when either the radicle or the shoot had emerged. Germination was expressed as the percentage of germinated seeds relative to the total number of seeds tested in each treatment. Root and shoot lengths were determined for the germinated seedlings.

### 2.4. Filter Paper Contact Test with Earthworms

Mature *Eisenia andrei* (Lumbricidae) originating from a laboratory culture established from specimens obtained from Jagiellonian University in Kraków, Poland, were maintained at the Department of Soil Science, Environmental Chemistry and Hydrology, University of Rzeszów, Poland. The initial culture was established using individuals identified on the basis of mitochondrial COI and nuclear 28S gene sequences [28,29]. Earthworms were reared at room temperature in containers filled with commercially available soil (Kronen Universalerde; pH (CaCl_2_) 5.5–6.5; N 200–450 mg L^−1^; P_2_O_5_ 200–400 mg L^−1^; K_2_O 300–500 mg L^−1^) and fed ad libitum with wheat flour as the sole food source.

Acute toxicity was assessed using a modified filter-paper contact test adapted from OECD [30]. Adult earthworms with a fully developed clitellum and a body mass of approximately 0.8 ± 0.1 g (RADWAG, Radom, Poland) were selected randomly from healthy individuals in good physiological condition. Before exposure, the earthworms were rinsed with water, gently dried on filter paper, and placed on moist filter paper to allow gut clearance.

Three independently prepared aqueous extracts of the composite material were tested at nominal concentrations of 50, 100, and 150 g L^−1^. For each concentration, 10 earthworms were tested individually in 10 separate 5 cm glass Petri dishes, each lined with Whatman No. 1 filter paper. The filter paper was moistened with 5 mL of the respective undiluted extract. The negative control consisted of 10 earthworms exposed individually under identical conditions to 5 mL of distilled water. All Petri dishes were covered and incubated at 20 ± 2 °C (BMT Medical Technology, Brno, Czech Republic) in darkness.

The same individuals were examined after 24 and 48 h of exposure. Mortality was defined as the absence of movement in response to gentle mechanical stimulation. In addition, visible morphological and behavioural abnormalities were recorded, including excessive mucus secretion, body swelling, constrictions, colour changes, surface lesions, coiling, abnormal posture, and reduced mobility. The experiment was performed once.

### 2.5. Bioluminescence Inhibition Assay

The acute toxicity of the aqueous extracts was assessed using the Aqua Science BioLight Toxy system (Aqua Science LLC, Newark, DE, USA) with BioLight Multi lyophilised bacterial reagent. The assay is based on the inhibition of bioluminescence emitted by the marine bacterium *Aliivibrio fischeri*. The BioLight reagents are manufactured and quality-controlled in accordance with the requirements of ISO 11348-3 [31] for tests using lyophilised bacteria.

The aqueous extracts prepared at nominal material concentrations of 50, 100, and 150 g L^−1^ were tested separately. Each undiluted extract was treated as the 100% test sample and subsequently subjected to serial 1:2 dilutions according to the instrument protocol. Sample salinity was adjusted using the reagents supplied with the test system, following the manufacturer’s instructions. The pH of the extracts was not adjusted before testing.

The lyophilised bacterial reagent was reconstituted with chilled reconstitution solution and conditioned in the instrument before exposure. Initial bioluminescence was measured before contact with the test sample. Subsequently, 0.9 mL of each test solution was transferred to the corresponding test vessel, and bioluminescence was measured after 5, 15, and 30 min of incubation. The test vessels and reading chamber were maintained at 15 °C during the assay. The BioLight Toxy system uses a photomultiplier tube to measure changes in bacterial light emission and reports toxicity as percentage effect or EC_50_, depending on the selected protocol.

The percentage inhibition of bacterial bioluminescence was calculated according to the procedure described in the BioLight Toxy Methods Manual [32]. As initial bioluminescence measurements were performed, the calculations were corrected for time-dependent changes in the bioluminescence of the negative control. The percentage inhibition was calculated as:

(1) $$Bioluminescence\;inhibition\;(\%)\;=\;\frac{G_{t}}{1\;+\;G_{t}}\;\times \;100$$

where *G_t_* is the luminescence inhibition factor at exposure time t, calculated as [32]:

(2) $$G_{t}\;=\;\frac{f_{t}\;\times \;I_{s0}}{I_{\mathrm{st}}}\;-\;1$$

The time-correction factor was calculated as:

*f_t_* = *I_βt_*/*I_β_*_0_ (3)

where *I_β_*_0_ and *I_βt_* are the luminescence intensities of the negative control before exposure and after time t, respectively; *I_s_*_0_ and *I_st_* are the corresponding luminescence intensities of the test sample; and *f_t_* is the time-correction factor accounting for changes in control luminescence. The EC_50_ was defined as the percentage concentration of the aqueous extract causing a 50% reduction in bacterial bioluminescence after 5, 15, or 30 min of exposure. EC_50_ values were derived from the concentration–response curves generated from the serial dilutions using BioLight Toxy software.

Toxicity units (*TU*) were calculated from the EC_50_ values, expressed as percentage concentrations of the aqueous extracts, using the following relationship [33]:

(4) $${TU}_{t}\;=\;\frac{100}{{EC}_{50,t}}$$

where *TU_t_* is the toxicity-unit value calculated for exposure time *t*, and *EC*_50,*t*_ is the percentage concentration of the extract causing 50% inhibition after 5, 15, or 30 min. Accordingly, the results are reported as *TU*_5_, *TU*_15_, and *TU*_30_. Higher *TU* values indicate greater acute toxicity.

### 2.6. Statistical Analysis

The experimental results are expressed as means and standard deviations (mean ± SD), calculated from replicate measurements. The standard deviation was used to characterise the variability of the experimental data.

Differences in *H. vulgare* root and shoot lengths among the control and extract treatments were assessed using one-way analysis of variance (ANOVA), followed by Tukey’s honestly significant difference (HSD) post hoc test for all pairwise comparisons of group means.

Spearman’s rank correlation analysis was used to assess monotonic relationships among selected physicochemical and biological variables. Correlation coefficients were visualised using a heat map.

Principal component analysis (PCA) was applied as an exploratory multivariate technique to identify the main response patterns and relationships among the analysed variables. Because the variables were expressed in different units, they were standardised prior to PCA using z-score transformation. Eigenvalues were used to determine the proportion of total variance explained by each principal component, while factor loadings were interpreted as measures of the relationships between the original variables and the principal components. Spearman rank correlations and principal component analysis were performed using the individual replicate-level observations rather than treatment means. Before PCA, variables measured on different scales were standardised to z-scores.

All statistical analyses were performed using Statistica 13.3 software (TIBCO Software Inc., Palo Alto, CA, USA). Statistical significance was accepted at α = 0.05.

## 3. Results and Discussion

### 3.1. Chemical Composition and pH-Dependent Leaching Behaviour

The solid composite contained 25.7% total organic carbon (TOC), reflecting the presence of the phenol–formaldehyde resin and cellulosic constituents. The concentrations of the targeted organic contaminants determined in the solid material are presented in Table 1. The total concentration of polycyclic aromatic hydrocarbons (PAHs) was 1.84 ± 0.42 mg kg^−1^ d.m., expressed as the analytical result together with its reported measurement uncertainty. Among the individual PAHs, only phenanthrene (1.02 ± 0.23 mg kg^−1^ d.m.) and naphthalene (0.21 ± 0.05 mg kg^−1^ d.m.) were quantified above their respective limits of quantification. The remaining individual PAHs, benzene, toluene, xylenes, ethylbenzene, and the analysed polychlorinated biphenyls were below the corresponding limits of quantification.

These results indicate that the targeted persistent and volatile organic contaminants occurred at low concentrations in the final composite. However, because neither the individual raw materials nor intermediate production stages were analysed, no conclusions can be drawn regarding changes in contaminant concentrations during manufacturing. Moreover, the total content of a compound in the solid material does not directly indicate its mobility or bioavailability during contact with water, environmental ageing, or end-of-life treatment. The generally low concentrations of the targeted organic contaminants are consistent with the broader observation that thermally stabilised carbonaceous or composite matrices may limit the mobility of some trace organic compounds, although the effectiveness of immobilisation depends strongly on matrix composition and exposure conditions [34].

The results of the aqueous-extract analyses are presented in Table 2. Under both extraction conditions, mercury, antimony, cadmium, molybdenum, and selenium remained below their respective limits of quantification. The acidic extraction condition selectively increased the release of several elements. Compared with the extract prepared at approximately pH 6.0, the concentrations of barium, chromium, zinc, copper, and lead were higher in the extract prepared at pH 3.5. The largest relative increases were observed for zinc, lead, and barium. In contrast, arsenic and nickel did not increase under acidic conditions. The increased release of selected metals under acidic conditions may be partly explained by proton-promoted dissolution of susceptible metal-bearing phases. For example, proton-promoted dissolution of a divalent metal oxide can be represented by the following general reaction [35]:

MO + 2H^+^ → M^2+^ + H_2_O (5)

where M represents a divalent metal. This equation is illustrative rather than a reaction confirmed for the investigated material, because the chemical forms of the released elements were not identified. The mobilisation of chromium and barium, in particular, cannot be inferred from this simplified oxide-dissolution model.

The phenol index showed the most pronounced pH-dependent change. It increased from 0.25 ± 0.06 mg L^−1^ at approximately pH 6.0 to 6.9 ± 1.7 mg L^−1^ at pH 3.5, corresponding to an approximately 28-fold increase. Because the composite contained a phenol–formaldehyde binding phase, this result suggests that acidic extraction conditions enhanced the mobilisation of phenolic compounds or other constituents responding in the phenol-index assay. However, this method does not identify individual compounds, and the detected response cannot be attributed to a specific phenol, monomer, oligomer, or degradation product without further chemical analysis. The acid–base equilibrium of phenol provides a simple illustration of the influence of pH on phenolic speciation [36]:

C_6_H_5_OH ⇌ C_6_H_5_O^−^ + H^+^ (6)

Because the pKa of phenol is approximately 10, the undissociated form predominates at both extraction pH values (approximately 6.0 and 3.5). Thus, phenol dissociation alone cannot explain the approximately 28-fold increase in the phenol index. The observed difference may reflect pH-dependent mobilisation of phenol-responsive constituents, but neither their identities nor the underlying release mechanisms were established by the present analyses. The mobility of phenolic constituents may also depend on their molecular structure and interactions with the polymer matrix, including partitioning and sorption processes [37]. Although chemical transformations of phenolic compounds have been reported under other extraction or processing conditions [38,39], those findings do not establish that comparable reactions occurred in the present experiments. Accordingly, the higher phenol-index response at pH 3.5 should be interpreted as evidence of increased release of assay-responsive constituents, not as proof of cleavage of the phenol–formaldehyde network or formation of a particular degradation product. Total dissolved solids increased from 970 to 1130 mg L^−1^ under acidic extraction conditions. Collectively, these findings demonstrate that the leaching behaviour of the composite depended strongly on extraction pH. Consequently, neutral-water extraction alone may underestimate the mobility of selected metals and phenolic constituents under acidic environmental conditions. From the perspective of circular-material design and end-of-life management, these results indicate that the suitability of the composite for outdoor use, contact with acidic media, or disposal under variable pH conditions should be evaluated using additional long-term leaching and ageing tests.

### 3.2. Response of S. marinoi to Composite Leachates

**Figure 2:**
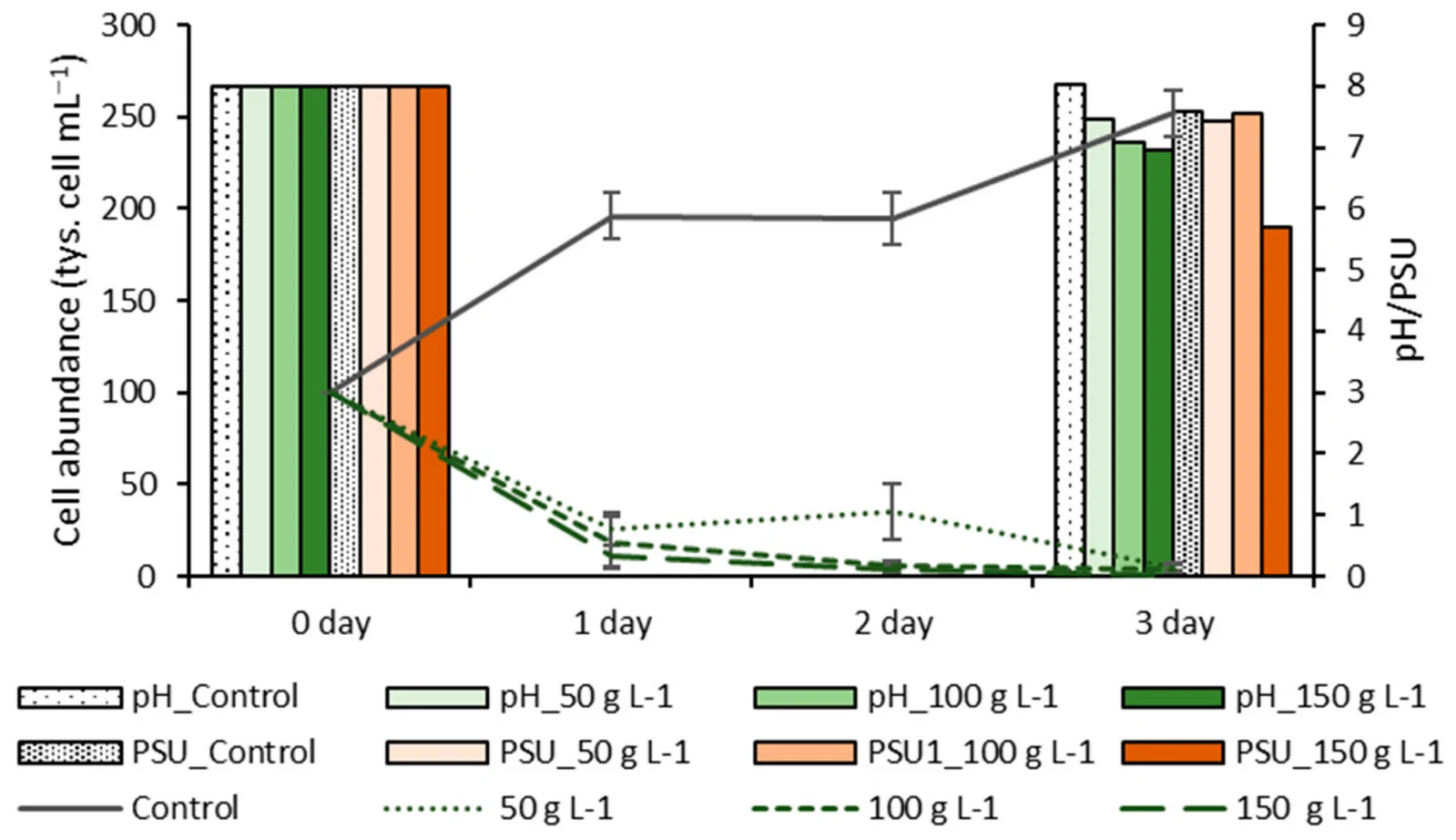
Response of *Skeletonema marinoi* to aqueous extracts of the composite: cell abundance during 3 days of exposure, expressed relative to the initial abundance at day 0; pH measured before exposure and after 3 days; and salinity measured before exposure and after 3 days. Extracts were prepared independently at nominal material concentrations of 50, 100, and 150 g L^−1^. Cell-abundance values are presented as mean ± SD from three independent culture flasks per treatment.

Exposure to the aqueous extracts resulted in a pronounced reduction in *S. marinoi* cell abundance, with the strongest effects observed at the highest extract concentrations and after the longest exposure period (Figure 2). Cell abundance was expressed relative to the initial abundance recorded at the beginning of the experiment. At 50 g L^−1^, the mean cell abundance corresponded to approximately 26% of the initial value after 1 day, 35% after 2 days, and 5% after 3 days. At 100 g L^−1^, the corresponding values were approximately 18%, 6%, and 4%, respectively. Exposure to the 150 g L^−1^ extract produced the greatest reduction, with cell abundance decreasing to approximately 11% after 1 day and 3% after 2 days; no cells were detected after 3 days.

In contrast, cell abundance in the control increased during the experiment, reaching approximately 196%, 194%, and 252% of the initial value after 1, 2, and 3 days, respectively. This divergence between the control and extract-exposed cultures demonstrates that the tested leachates substantially impaired diatom population development under the applied screening conditions. Among the organisms included in the test battery, *S. marinoi* showed the strongest biological response, indicating a particularly high sensitivity of this aquatic primary producer to substances released from the composite.

The pH and salinity measurements provide additional information on changes in the exposure media during the test (Figure 2). After 3 days, pH decreased with increasing extract concentration, from 8.03 in the control to 7.45, 7.09, and 6.95 in the 50, 100, and 150 g L^−1^ treatments, respectively. Salinity remained close to the initial value in the control and in the 50 and 100 g L^−1^ treatments but decreased to 5.7 PSU in the 150 g L^−1^ extract. These changes may have contributed to the response observed at the highest concentration. However, the available data do not allow the effects of pH and salinity to be separated from those of the chemical constituents released from the composite.

The chemical analysis identified several groups of compounds that may be relevant to the observed algal response. Polycyclic aromatic hydrocarbons are recognised as potential stressors in both marine and freshwater ecosystems and may affect the growth, photosynthetic activity, biomass production, and metabolism of aquatic microorganisms [40,41]. The composite contained a total PAH concentration of 1.84 ± 0.42 mg kg^−1^ d.m., including quantifiable concentrations of phenanthrene and naphthalene. Nevertheless, these values represent the total content in the solid material and cannot be directly translated into the concentrations to which the algae were exposed in the aqueous extracts. Therefore, the pronounced inhibition of S. marinoi cannot be attributed solely to the measured PAHs. Previous studies have shown that exposure to PAHs may alter algal and cyanobacterial community structure and, through changes in primary-producer abundance, indirectly affect higher trophic levels [42].

The aqueous extracts also contained barium, zinc, and copper, although their measured concentrations were relatively low. Organisms have element-specific tolerance ranges, and both deficiency and excess of essential elements may disrupt physiological processes and inhibit growth [43]. Zinc and copper are required in trace amounts for enzymatic activity and cellular metabolism, but they become toxic when their bioavailable concentrations exceed species-specific tolerance thresholds [44]. In the present study, the concentrations measured in the extract were substantially lower than the 96 h EC_50_ reported for zinc in the marine diatom Amphora coffeaeformis [45]. This comparison should, however, be interpreted cautiously because toxicity depends on species, exposure duration, water chemistry, metal speciation, and interactions with other leachate constituents.

Barium was also detected in the aqueous extracts. Although the measured concentration alone does not indicate acute toxicity, some protists, including diatoms, can accumulate barium through processes associated with calcium transport and intracellular mineral formation [46]. The environmental relevance of this mechanism for the tested composite cannot be established from the present short-term experiment, but it indicates that low aqueous concentrations do not necessarily exclude intracellular uptake during prolonged exposure.

The strong response of *S. marinoi* contrasted with the limited effects observed in the terrestrial test organisms. This difference demonstrates that the biological effects of the extracts were taxon-dependent and that apparent tolerance in one organism cannot be extrapolated to other ecological compartments. In addition, the relatively low concentrations of the targeted contaminants were insufficient to explain the magnitude of the observed algal response. The toxicity may therefore have resulted from the combined action of several constituents, unidentified leachables, or changes in the physicochemical properties of the exposure medium.

Potential contributors may include low-molecular-weight phenolic constituents, residual monomers or oligomers associated with the phenol–formaldehyde resin, aldehydes, transformation products, or other substances not included in the targeted analytical programme. However, these possibilities remain hypotheses and cannot be confirmed on the basis of the present data. The discrepancy between targeted chemical measurements and the pronounced biological response highlights the value of effect-based testing and supports the use of non-target screening and toxicity identification evaluation to identify the compounds or mixtures responsible for the observed inhibition.

### 3.3. Germination and Early Seedling Growth of H. vulgare

**Figure 3:**
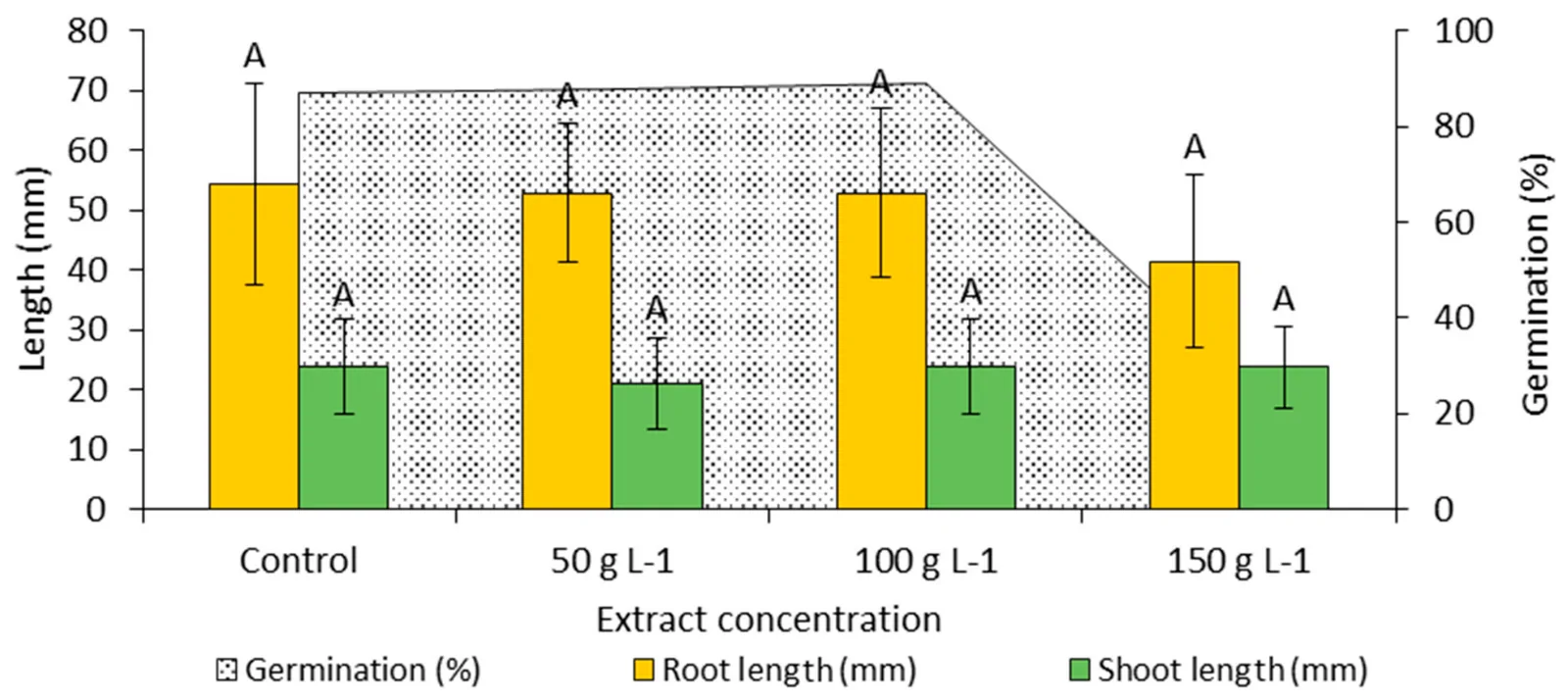
Effects of aqueous extracts of the composite on *Hordeum vulgare* after 72 h of exposure: germination; root length; and shoot length. Extracts were prepared independently at nominal material concentrations of 50, 100, and 150 g L^−1^. Root and shoot lengths are presented as mean ± SD from three independent Petri dishes per treatment. Identical letters A above Root length and Shoot length indicate no statistically significant differences among treatments (*p* > 0.05).

Exposure to the aqueous extracts produced a concentration-dependent effect on *Hordeum vulgare* germination (Figure 3). Germination in the 50 and 100 g L^−1^ treatments remained comparable to that in the control, whereas the 150 g L^−1^ extract caused a marked reduction. This pattern suggests the presence of an effect threshold above which the soluble fraction released from the composite interfered with seed germination. However, because the extracts contained a complex mixture of inorganic ions and organic constituents, the observed response cannot be attributed to any individual component on the basis of the present data.

The inhibition recorded at the highest extract concentration may have resulted from the combined action of several dissolved constituents rather than from a single compound. The extract contained, among other components, chlorides and sulfates, which may contribute to osmotic or ionic stress when present in combination with other soluble substances. Similar mixture-dependent effects were reported by Seal et al. [47], who observed stronger inhibition of *Suaeda maritima* germination under simultaneous exposure to multiple mineral salts than under exposure to individual salts. Nevertheless, differences in plant species, salt composition, and exposure conditions mean that this comparison should be treated as supporting context rather than as direct evidence of the mechanism responsible for the response of *H. vulgare.*

Root and shoot lengths did not differ significantly among the control and extract treatments (*p* > 0.05), although a slight reduction in root length was observed at 150 g L^−1^ (Figure 3). Shoot length remained relatively stable across treatments. Under the applied 72 h exposure conditions, germination was therefore more responsive to the highest extract concentration than early seedling elongation.

The absence of statistically significant differences in root and shoot lengths should not be interpreted as evidence that the released constituents were beneficial to plants. Rather, it indicates that their combined concentrations did not exceed the short-term effect threshold for these growth endpoints under the experimental conditions. Some of the inorganic constituents detected in the extracts, particularly sulfate, are essential for normal plant metabolism at appropriate concentrations. Sulfur is required for the biosynthesis of sulfur-containing amino acids, proteins, glutathione, and other compounds involved in antioxidant defence and cellular metabolism [48]. In *H. vulgare*, sulfate deficiency has been associated with reduced cysteine, glutathione, and soluble protein levels, as well as increased adenosine 5′-phosphosulfate reductase activity and activation of compensatory responses to sulfur limitation [49]. However, the physiological importance of sulfate does not demonstrate that the sulfate present in the extracts improved seedling development, particularly because the exposure involved a complex mixture of substances and no nutrient-response experiment was conducted.

Plant responses to inorganic ions also depend strongly on concentration, chemical form, exposure duration, and genotype. This is illustrated by the response of cereals to fluoride. Pelc et al. [50] reported that high fluoride concentrations inhibited germination, restricted root growth, and altered catalase activity in *Triticum aestivum*, whereas at lower concentrations, the magnitude of the response depended substantially on cultivar. This supports the interpretation that the absence of significant root and shoot inhibition in the present study may reflect both the relatively short exposure period and the tolerance of the tested *H. vulgare* seed material, rather than the complete absence of biologically active constituents.

Because germination and seedling elongation represent distinct physiological processes, their differing responses further support the use of multiple endpoints in the phytotoxicity assessment of waste-derived polymer composites. The marked response of germination at 150 g L^−1^, despite the lack of significant effects on root and shoot length, indicates that reliance on a single growth endpoint could underestimate short-term phytotoxic effects.

### 3.4. Acute Toxicity Response of the Earthworm E. andrei

Exposure to the aqueous extracts prepared at 50, 100, and 150 g L^−1^ resulted in 100% survival of *Eisenia andrei* after both 24 and 48 h (Table 3). Survival in the negative control was also 100%. No visible morphological or behavioural abnormalities were observed during the exposure period. The earthworms retained normal colour, body turgor, and mobility and showed no excessive mucus secretion, swelling, constrictions, surface lesions, coiling, or abnormal posture. Accordingly, none of the tested extracts produced detectable lethal or externally visible sublethal effects under the applied screening conditions.

The modified filter-paper contact test provides a rapid assessment of short-term toxicity resulting from direct contact with an aqueous extract. Acute contact tests and longer-term soil-based assays may produce different outcomes because they differ in exposure route, test-medium properties, exposure duration, and the biological endpoints considered. In earthworm studies, these endpoints may include survival, changes in body mass, reproduction, and biochemical or cellular indicators of stress [51]. Therefore, the absence of mortality and visible abnormalities in the present test should not be interpreted as evidence of environmental safety under all terrestrial exposure scenarios.

The observed lack of acute contact toxicity is broadly consistent with reports showing limited short-term effects of some polymer-based materials on earthworms [52]. Similar low acute responses have also been reported where the tested materials released only a limited water-soluble fraction under the applied conditions [53]. However, such comparisons should be interpreted cautiously because the toxicity of polymeric and composite materials depends on their chemical composition, additives, ageing state, particle size, degradation products, and the characteristics of the exposure medium.

The present findings indicate that the aqueous extracts did not contain concentrations of readily bioavailable constituents sufficient to cause mortality or externally visible stress responses in *E. andrei* during the 48 h exposure period. They do not exclude effects resulting from prolonged exposure, soil-mediated transformations, dietary uptake, ingestion of particles, or contact with weathered and fragmented material. The filter-paper system does not reproduce sorption to mineral and organic soil phases, transformation of released substances, feeding exposure, particle ingestion, or the progressive release of constituents from aged composite residues.

A more comprehensive assessment of potential terrestrial effects would therefore require chronic soil-based studies incorporating additional endpoints, such as changes in body mass, cocoon production, reproduction, juvenile development, and biochemical or cellular biomarkers. Such tests would also allow the effects of ageing, fragmentation, and particle ingestion to be considered. The present result should consequently be regarded as a screening-level indication of low acute contact toxicity rather than as a complete evaluation of the environmental safety of the composite for land application, outdoor use, or disposal.

The lack of an acute response in *E. andrei* contrasts with the pronounced inhibition observed in *S. marinoi.* This difference demonstrates that the biological effects of the composite extracts were taxon- and compartment-dependent. A leachate that caused no detectable acute effect in a terrestrial invertebrate substantially impaired the development of an aquatic primary producer. Consequently, the environmental assessment of waste-derived polymer composites should be based on a multispecies approach and should consider the most sensitive environmentally relevant endpoint rather than relying on the response of a single, relatively tolerant organism.

### 3.5. Toxicity Assessment Based on Bioluminescence Inhibition of A. fischeri

**Figure 4:**
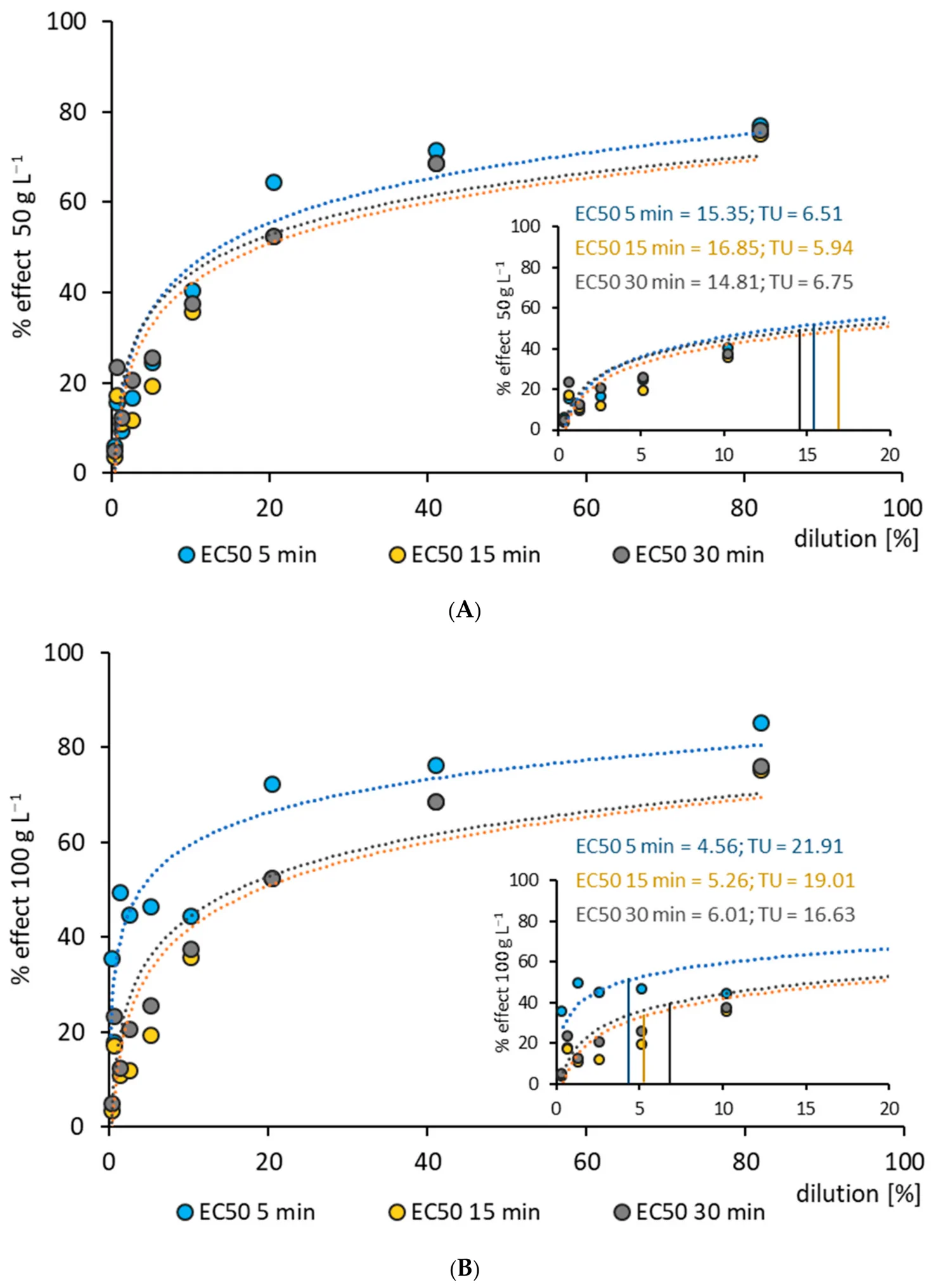

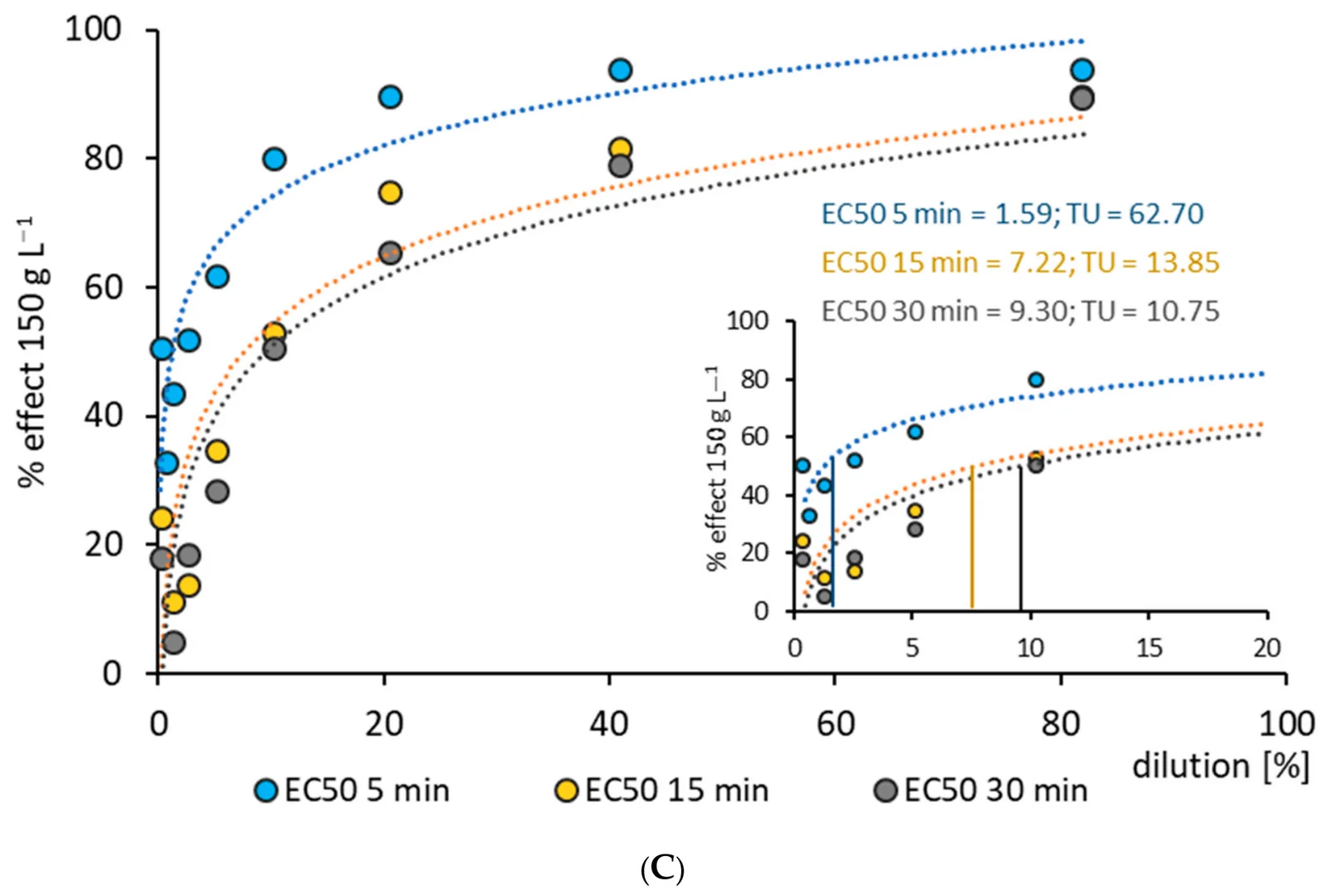
Concentration–response relationships for inhibition of *Aliivibrio fischeri* bioluminescence after 5, 15, and 30 min of exposure to aqueous composite extracts prepared at nominal material concentrations of (**A**) 50 g L^−1^, (**B**) 100 g L^−1^, and (**C**) 150 g L^−1^. Insets show the EC_50_ estimates used to calculate toxicity units (TU).

Bioluminescence inhibition of *Aliivibrio fischeri* increased with extract concentration (Figure 4). Because bacterial light emission reflects cellular metabolic activity, its inhibition is widely used as a sensitive indicator of acute toxicity and may occur without direct cell mortality [54]. The 50 g L^−1^ extract showed the lowest acute toxicity, with an EC_50_ of 15.35% after 5 min and TU values of 6.51, 5.94, and 6.75 after 5, 15, and 30 min, respectively. The 100 g L^−1^ extract produced a stronger response, with TU values decreasing from 21.93 after 5 min to 19.01 after 15 min and 16.64 after 30 min. The 150 g L^−1^ extract showed the highest toxicity at 5 min (TU_5_ = 62.89).

For the 100 and 150 g L^−1^ extracts, the decrease in TU with exposure time indicates that the strongest measurable effect occurred during the initial incubation period. Similar time-dependent changes in bacterial toxicity responses have been associated with variations in contaminant bioavailability, physicochemical transformation of toxic constituents, adsorption, and physiological adjustment of the test organism during exposure [55,56]. *A. fischeri* can exhibit physiological responses that enable it to remain metabolically active under exposure to a range of environmental contaminants, which contributes to its usefulness in toxicity monitoring [57,58]. Nevertheless, the present results do not allow reduced bioavailability, chemical transformation, or bacterial adaptation to be distinguished experimentally. The decline in TU should therefore be interpreted as evidence of a time-dependent change in the apparent toxicity of the leachate rather than as proof of a specific detoxification or adaptation mechanism.

A slightly different pattern was observed for the 50 g L^−1^ extract. Toxicity decreased between 5 and 15 min but increased again after 30 min, as indicated by TU values of 6.51, 5.94, and 6.75, respectively. This small temporal variation may reflect changes in the availability or activity of individual leachate constituents, bacterial physiological adjustment, or transformation of dissolved compounds during incubation. However, because the differences were modest and no compound-specific measurements were conducted during the assay, the formation of more toxic transformation products or exhaustion of an adaptive response cannot be confirmed.

The targeted metal concentrations in the near-neutral extract were low. Literature data indicate that the toxicity of metal-containing mixtures cannot always be predicted directly from nominal concentrations because biological effects depend on speciation, interactions among constituents, and physicochemical properties of the exposure medium [59,60]. Accordingly, the measured concentrations of the selected metals alone were unlikely to explain the magnitude of the observed bioluminescence inhibition, although their contribution as part of a complex mixture cannot be excluded.

The phenol index increased from 0.25 ± 0.06 mg L^−1^ at approximately pH 6.0 to 6.9 ± 1.7 mg L^−1^ at pH 3.5. Changes in pH may affect the apparent concentration and behaviour of phenolic compounds through modifications in solubility, partitioning, oxidative stability, adsorption, biodegradation, extraction efficiency, and analytical response [33]. Phenolic compounds are plausible contributors to bacterial luminescence inhibition, but their toxicity depends strongly on molecular structure, including the nature and position of substituents on the aromatic ring. Certain substituent groups may substantially increase toxicity, particularly under acidic conditions [61].

Previous studies have reported substantial variation in the toxicity of individual phenolic compounds to *A. fischeri*. Catechol and other phenolic constituents may inhibit bacterial bioluminescence at relatively low concentrations, although the reported effect ranges depend on compound identity and test conditions [62]. More generally, increasing concentrations of phenolic compounds are frequently associated with lower EC_50_ values and therefore greater apparent toxicity in bioluminescence assays [63]. Mixtures of structurally different phenolic compounds may further modify the observed response through additive, antagonistic, or synergistic interactions [64].

However, the phenol index is a group parameter and does not identify catechol or any other individual compound. Therefore, catechol cannot be identified as the cause of toxicity on the basis of the present results. Similarly, the toxicity measured in the *A. fischeri* assay cannot be directly linked to the phenol index obtained under acidic extraction, because the bioassays were performed using separately prepared extracts and the available data do not establish the concentrations of individual phenolic compounds in the test media.

The discrepancy between the relatively low concentrations of targeted metals and the measurable inhibition of bacterial bioluminescence suggests that unidentified organic constituents, transformation products, or mixture effects may have contributed to the observed response. Potential contributors may include phenolic compounds, residual low-molecular-weight constituents associated with the phenol–formaldehyde resin, or other leachable compounds not included in the targeted analytical programme. The *A. fischeri* response therefore supports the need for compound-specific and non-target analyses, particularly after accelerated ageing, repeated leaching, or acidic exposure. Such information would help determine whether the material can be safely reused, incorporated into another product, or requires controlled end-of-life management.

### 3.6. Relationships Between Physicochemical and Ecotoxicological Parameters

**Figure 5:**
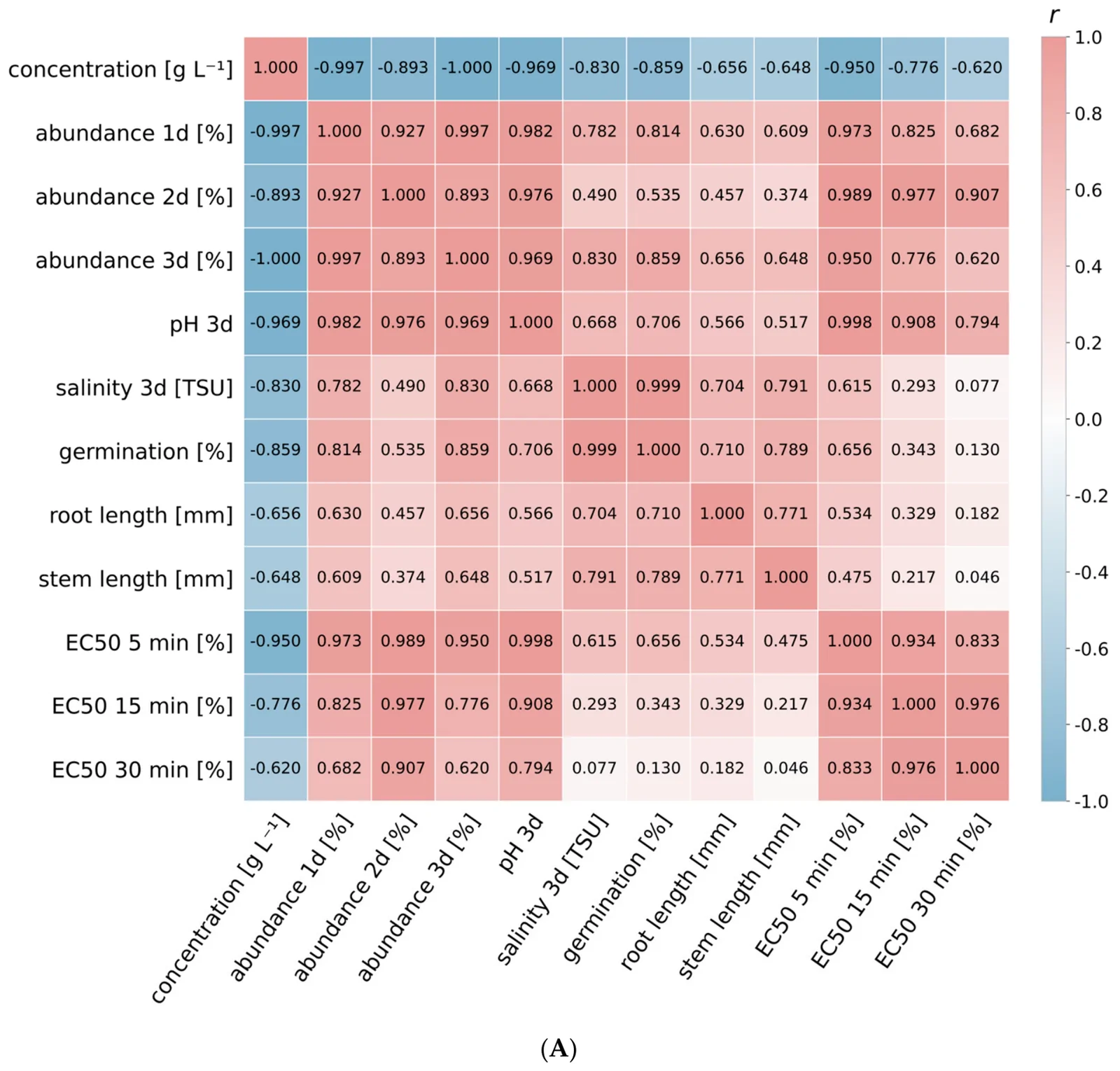

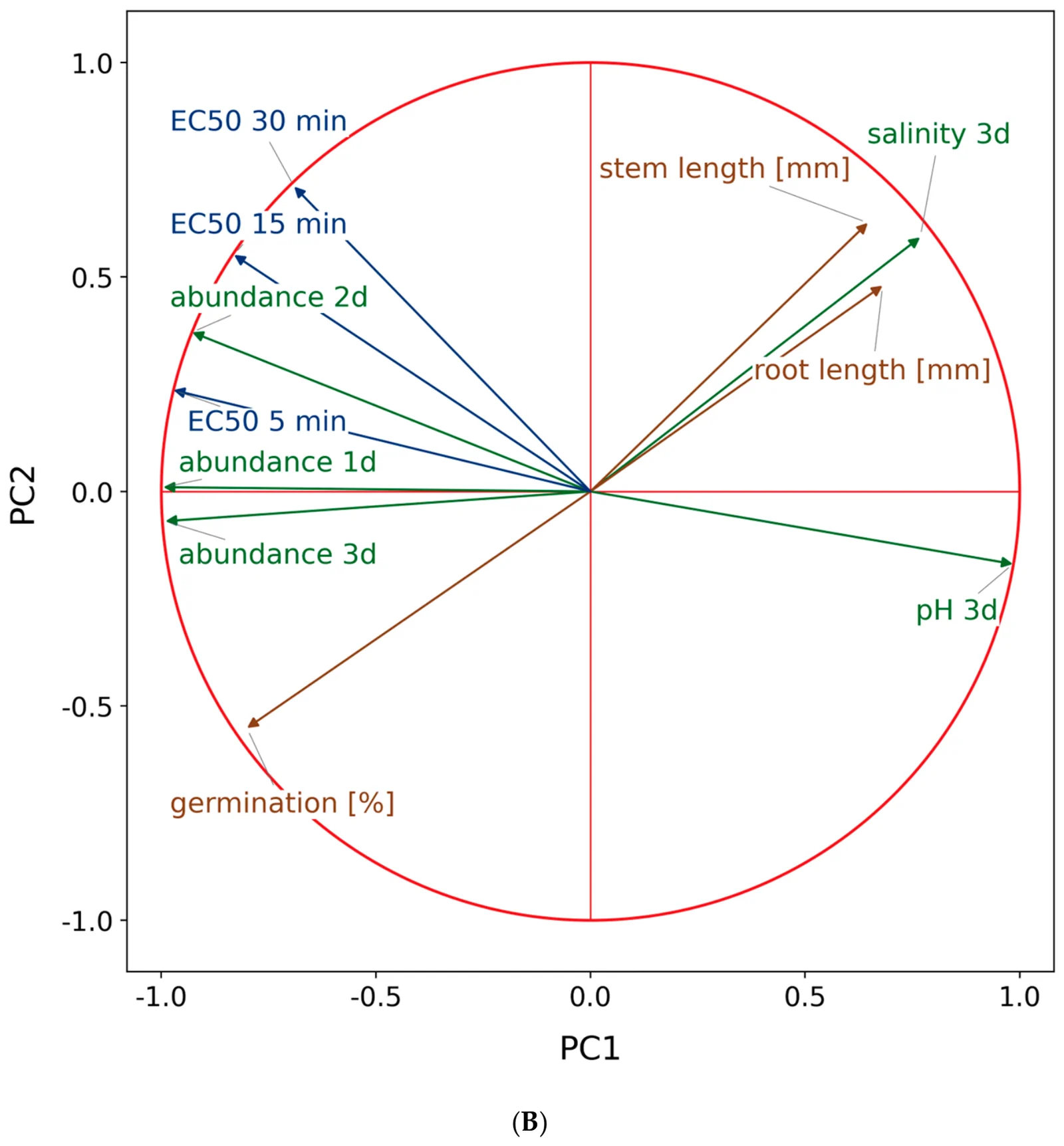
Multivariate relationships among physicochemical parameters and ecotoxicological responses to aqueous composite extracts prepared at nominal material concentrations of 50, 100, and 150 g L^−1^: (**A**) Spearman rank-correlation matrix and (**B**) principal component analysis (PCA) biplot for PC1 (72.97%) and PC2 (21.04%). In the PCA biplot, arrow colours distinguish different groups of variables: blue arrows represent ecotoxicological responses expressed as EC50 values, green arrows indicate physicochemical parameters (pH and salinity) and organism abundance, and brown arrows represent plant growth and germination parameters. EC_50_, median effective concentration; PSU, practical salinity unit.

Extract concentration was negatively associated with most biological-performance endpoints, consistent with the overall increase in adverse effects across the tested concentration gradient (Figure 5A). Germination, root length, and shoot length were positively correlated, indicating a coherent plant-response pattern. *S. marinoi* cell abundance was also positively associated with the plant-performance variables and with the EC_50_ values obtained for *A. fischeri*. Because higher EC_50_ values indicate lower apparent toxicity, these relationships show that greater biological performance generally coincided with a weaker toxic response in the bacterial assay.

The correlation analysis was performed using individual replicate-level observations rather than treatment means. Nevertheless, because the analysis combined endpoints obtained from different organisms, exposure durations, and measurement scales, the observed relationships should be interpreted as exploratory associations rather than as evidence of shared biological mechanisms or causal relationships.

The principal component analysis, performed using individual replicate-level observations, provided a descriptive synthesis of the relationships among the measured endpoints. PC1 explained 72.97% of the total variance, whereas PC2 explained a further 21.04%, resulting in a cumulative explained variance of 94.01% (Figure 5B). PCA is commonly used to reduce the dimensionality of multivariable datasets and to identify the variables contributing most strongly to the dominant patterns of variation. However, the resulting components reflect the structure of the analysed dataset and should not automatically be assigned a causal interpretation.

Variables describing organism performance, including *S. marinoi* cell abundance and *H. vulgare* germination, contributed strongly to PC1 and were positioned opposite the extract-concentration gradient. This distribution was consistent with the overall decline in biological performance at increasing extract concentrations. Root and shoot lengths were associated with the other plant-response variables, although shoot length contributed to both PC1 and PC2, indicating that its response was not explained exclusively by the dominant concentration-related pattern.

The pH and salinity values measured during the *S. marinoi* assay showed different contributions to the principal components than the primary biological endpoints. This indicates that changes in the physicochemical properties of the exposure media and changes in organism performance did not follow identical patterns. Although pH and salinity may have contributed to the response observed in the algal assay, the PCA does not allow their influence to be separated from that of the chemical constituents released from the composite.

The *A. fischeri* endpoints contributed to both principal components, consistent with the distinct time-dependent pattern observed in the bioluminescence assay. The responses recorded after 5 and 15 min were more strongly associated with PC1, whereas the 30 min response contributed more prominently to PC2. This differentiation reflects variation in the apparent toxicity of the extracts over the incubation period, but it does not identify the chemical transformations, changes in bioavailability, or bacterial physiological responses responsible for that pattern.

The PCA was therefore used as an exploratory summary of the multispecies test battery rather than as a predictive model of environmental risk. Taken together, the correlation and PCA results demonstrate that the biological response was not uniform across taxa and that the environmental interpretation depended strongly on the selected endpoint. The multivariate analysis does not identify the chemical cause of toxicity, but it reinforces the value of combining organisms representing different trophic levels and environmental compartments when screening waste-derived polymer composites.

### 3.7. Implications for Sustainable Composite Design and End-of-Life Strategies

The investigated board demonstrates a relevant circular-economy pathway because it incorporates industrial glass-fibre and polymer residues into a new functional product. However, the results also show that waste diversion and mechanical performance are only part of sustainability assessment. For thermoset-based composites, where repeated melt reprocessing is not available, decisions on reuse, cascading applications, recycling, or disposal should account for the stability of the polymer matrix and the properties of the released fraction. The marked pH dependence of metal and phenolic release indicates that exposure conditions can change the environmental profile of the material and should be considered during product design and application selection.

The present screening supports several practical safe-by-design priorities: reducing the mobile low-molecular-weight fraction of the phenol–formaldehyde system; verifying matrix stability after water uptake and acidic exposure; selecting applications that minimise prolonged contact with aquatic environments; and assessing whether coatings, formulation changes, or additional curing reduce leachate toxicity. At end-of-life, intact reuse or controlled cascading may be preferable to pathways that increase surface area, generate particles, or expose the composite to aggressive chemical conditions. These implications are derived from short-term laboratory tests and should be validated under ageing, weathering, repeated-leaching, and realistic service scenarios.

This study does not constitute a full life-cycle assessment and does not compare alternative recycling technologies. Its contribution is narrower but complementary: it provides environmental-performance evidence that can be incorporated into life-cycle thinking and into the selection of technically and environmentally appropriate end-of-life routes. In this sense, ecotoxicological screening functions as an early decision tool for sustainable composite development rather than as a final regulatory risk assessment.

## 4. Conclusions

The combined chemical and ecotoxicological assessment of the waste-derived phenol–formaldehyde/glass-fibre composite led to the following conclusions:

1. Leaching behaviour depended on extraction pH. Compared with extraction at approximately pH 6.0, extraction at pH 3.5 increased the release of Ba, Cr, Zn, Cu, and Pb. The phenol index increased approximately 28-fold, from 0.25 ± 0.06 to 6.9 ± 1.7 mg L^−1^. These findings indicate that near-neutral extraction alone may underestimate the release of selected constituents under acidic conditions.
2. *Skeletonema marinoi* exhibited the most pronounced response within the test battery. Exposure to the composite extracts substantially reduced cell abundance, and no living cells were detected after three days at the highest nominal material loading of 150 g L^−1^. Changes in exposure-medium pH and salinity may have contributed to this response, but their effects could not be separated from those of the released constituents.
3. The *Aliivibrio fischeri* assay confirmed the acute biological activity of the extracts. After 5 min, toxicity increased with nominal material loading, with toxicity units of 6.51, 21.93, and 62.89 for the 50, 100, and 150 g L^−1^ extracts, respectively. The subsequent decrease in toxicity for the two higher loadings demonstrated a time-dependent response, without establishing a specific detoxification or adaptation mechanism.
4. Short-term effects on the terrestrial organisms were limited to the endpoints examined. *Hordeum vulgare* germination decreased at the highest extract loading, whereas root and shoot lengths did not differ significantly among treatments. *Eisenia andrei* survival remained at 100%, with no visible morphological or behavioural abnormalities after 24 and 48 h. These findings do not establish the absence of chronic effects under soil exposure.
5. The targeted chemical analyses did not establish the constituents responsible for the observed biological responses. Unidentified leachables, mixture effects, and changes in exposure-medium properties remain possible contributors. Combining chemical characterisation with multiple biological endpoints therefore provided a more informative screening assessment than either approach alone.
6. Incorporating industrial residues into a functional composite does not, by itself, demonstrate environmental safety or overall sustainability. Application selection and end-of-life management should account for pH-dependent release and the responses of sensitive aquatic organisms. The integrated assessment used here can support early safe-by-design decisions.

The findings are restricted to the applied laboratory extraction and short-term exposure conditions. Further assessment should include ageing, repeated leaching, particle release, chronic aquatic and soil-based tests, and identification of biologically active constituents before broader conclusions about environmental safety can be drawn.

Overall, this study highlights the limitations of assessing the environmental suitability of waste-derived polymer composites solely based on recycled content and targeted chemical analyses. The findings underscore the importance of integrating pH-dependent leaching assessments with ecotoxicological testing to identify potential environmental hazards that may not be apparent from chemical measurements alone. This integrated approach can support material optimisation, application selection, and end-of-life management, although further investigations under realistic, long-term environmental exposure conditions are required before definitive conclusions regarding environmental safety can be drawn.

## Figures and Tables

**Table 1:** Targeted organic contaminants determined in the waste-derived composite board.

Compound or Group	Result ± Measurement Uncertainty (mg kg^−1^ d.m.)
Polycyclic Aromatic Hydrocarbons (PAHs)	1.84 ± 0.42
Chrysene	<0.010
Fluoranthene	<0.020
Benzo(a)pyrene	<0.010
Benzo(b)fluoranthene	<0.020
Benzo(k)fluoranthene	<0.010
Dibenzo(a,h)anthracene	<0.020
Indeno(1,2,3-cd)pyrene	<0.010
Benzo(a)anthracene	<0.010
Phenanthrene	1.02 ± 0.23
Benzo(ghi)perylene	<0.020
Naphthalene	0.21 ± 0.05
Anthracene	<0.010
Volatile Aromatic Hydrocarbons (VAHs)	<0.020
Benzene	<0.020
Toluene	<0.020
Xylenes	<0.020
Ethylbenzene	<0.020
Polychlorinated Biphenyls (PCBs)	<0.020

**Table 2:** Selected inorganic, carbon-related, and phenolic parameters determined in aqueous extracts of the waste-derived composite board prepared at approximately pH 6.0 and pH 3.5.

Parameter	Extract at pH 6.0	Extract at pH 3.5	Unit
Inorganic ions and dissolved carbon			
Chlorides	15.5	n.d.	mg kg^−1^ d.m.
Sulfates	39.9	n.d.	mg kg^−1^ d.m.
Fluorides	2.08	n.d.	mg kg^−1^ d.m.
Dissolved organic carbon (DOC)	889	n.d.	mg kg^−1^ d.m.
Metals and metalloids			
Mercury (Hg)	<0.0007	<0.0007	mg kg^−1^ d.m.
Antimony (Sb)	<0.001	<0.001	mg kg^−1^ d.m.
Arsenic (As)	0.009	0.002	mg kg^−1^ d.m.
Barium (Ba)	0.162	0.849	mg kg^−1^ d.m.
Chromium (Cr)	<0.001	0.008	mg kg^−1^ d.m.
Zinc (Zn)	0.092	2.83	mg kg^−1^ d.m.
Cadmium (Cd)	<0.001	<0.001	mg kg^−1^ d.m.
Copper (Cu)	0.070	0.155	mg kg^−1^ d.m.
Molybdenum (Mo)	<0.001	<0.001	mg kg^−1^ d.m.
Nickel (Ni)	0.008	0.006	mg kg^−1^ d.m.
Lead (Pb)	0.004	0.071	mg kg^−1^ d.m.
Selenium (Se)	<0.001	<0.001	mg kg^−1^ d.m.
General indicators			
Total dissolved solids (TDS)	970	1130	mg L^−1^
Phenol index/volatile phenols	0.25 ± 0.06	6.9 ± 1.7	mg L^−1^

n.d.—not determined; d.m.—dry matter.

**Table 3:** Survival of *Eisenia andrei* after 24 and 48 h of exposure to aqueous extracts of the composite prepared independently at nominal material concentrations of 50, 100, and 150 g L^−1^. Ten earthworms were tested individually per treatment and in the negative control.

Treatment	Survival After 24 h (%)	Survival After 48 h (%)
Negative control	100	100
50 g L^−1^ extract	100	100
100 g L^−1^ extract	100	100
150 g L^−1^ extract	100	100

## Data Availability

The data presented in this study are available from the corresponding author upon reasonable request.
